# Improved Production of* Aspergillus usamii* endo-*β*-1,4-Xylanase in* Pichia pastoris* via Combined Strategies

**DOI:** 10.1155/2016/3265895

**Published:** 2016-03-15

**Authors:** Jianrong Wang, Yangyuan Li, Danni Liu

**Affiliations:** Guangdong VTR Bio-Tech Co., Ltd., Zhuhai, Guangdong 519060, China

## Abstract

A series of strategies were applied to improve expression level of recombinant endo-*β*-1,4-xylanase from* Aspergillus usamii* (*A. usamii*) in* Pichia pastoris* (*P. pastoris*). Firstly, the endo-*β*-1,4-xylanase (*xynB*) gene from* A. usamii* was optimized for* P. pastoris* and expressed in* P. pastoris*. The maximum xylanase activity of optimized (*xynB-opt*) gene was 33500 U/mL after methanol induction for 144 h in 50 L bioreactor, which was 59% higher than that by wild-type (*xynB*) gene. To further increase the expression of* xynB-opt*, the* Vitreoscilla hemoglobin* (*VHb*) gene was transformed to the recombinant strain containing* xynB-opt*. The results showed that recombinant strain harboring the* xynB-opt* and* VHb* (named X33/*xynB-opt-VHb*) displayed higher biomass, cell viability, and xylanase activity. The maximum xylanase activity of X33/*xynB-opt-VHb* in 50 L bioreactor was 45225 U/mL, which was 35% and 115% higher than that by optimized (*xynB-opt*) gene and wild-type (*xynB*) gene. Finally, the induction temperature of X33/*xynB-opt-VHb* was optimized in 50 L bioreactor. The maximum xylanase activity of X33/*xynB-opt-VHb* reached 58792 U/mL when the induction temperature was 22°C. The results presented here will greatly contribute to improving the production of recombinant proteins in* P. pastoris*.

## 1. Introduction

Xylan, the major hemicellulose component in plant cell wall and the most abundant renewable hemicellulose, is a heterogeneous polysaccharide consisting of a *β*-1,4-linked D-xylose backbone [1]. Xylan occupies one-third of the overall plant carbohydrate and is the second most prevalent biomass after cellulose in nature [2]. Xylanase (EC 3.2.1.8) can hydrolyze xylan into xylooligosaccharides and D-xylose. Xylanases have generated considerable research interest and are becoming a major group of industrial enzymes. Xylanases have wide commercial applications in industrial processes, such as the paper and pulp industry, the foodstuff industry, the feed industry, and the energy industry [3, 4]. In recent years, increasing numbers of xylanases have been identified and characterized from various microorganisms, such as bacteria and fungi [5]. Among microbial sources, the filamentous fungi* Aspergillus* are especially interesting as they secrete these enzymes into the medium and their xylanase activities are higher than those produced by other microorganisms [6–8]. In previous studies, an endo-*β*-1,4-xylanase with high specific activity and good enzymatic properties was isolated from* A. usamii*. Furthermore, the gene encoding endo-*β*-1,4-xylanase from* A. usamii* was cloned and expressed in* P. pastoris* [9, 10]. However, the low expression level does not allow the recombinant xylanase to be applied practically and economically in industry. For commercial exploitation of the recombinant* A. usamii* endo-*β*-1,4-xylanase, it is essential to achieve high yield of the protein.

*P. pastoris* is now widely used for heterologous production of recombinant proteins. As a single-celled microorganism,* P. pastoris* has been proved as a highly successful system for variety of recombinant proteins. There are many advantages in this expression system such as high level expression, efficient extracellular protein secretion, proper protein folding, posttranslational modifications, and the potential to high cell density fermentation [11, 12]. Due to the wide use of this system, many strategies have been developed to further improve expression level of heterologous protein in* P. pastoris*, including codon optimization, intracellular coexpression of* Vitreoscilla hemoglobin* (VHb), high heterologous gene copy number, altering the secretion signal peptide in expression vector, high efficient transcriptional promoters, and cultivation optimization [13–15]. However, there is little report about integrating these optimization methods as a whole to optimize gene expression.

In order to improve production of* A. usamii* endo-*β*-1,4-xylanase in* P. pastoris*, the* xynB* from* A. usamii* was firstly expressed in* P. pastoris*. To further improve the production of recombinant* A. usamii* endo-*β*-1,4-xylanase, we combined codon optimization, intracellular coexpression of VHb, and optimization of induction temperature as a whole to optimize* xynB* expression in* P. pastoris*. To our knowledge, this is the first report to improve* A. usamii* endo-*β*-1,4-xylanase production in* P. pastoris* by integrating these three optimization methods. The results presented here will greatly contribute to improving the production of recombinant proteins in* P. pastoris *and offer a greater value in various industrial applications.

## 2. Materials and Methods

### 2.1. Strains, Plasmids, Reagents, and Media

The* P. pastoris* strain X33 and the expression vector (pPIC3.5K and pPICZ*α*A) were purchased from Invitrogen (Carlsbad, CA, USA). The* E. coli* strain Top 10 is routinely conserved in our laboratory. Restriction enzymes, T4-DNA ligase, and Pfu DNA polymerase were purchased from Sangon Biotech (Shanghai, China). All other chemicals used were analytical grade reagents unless otherwise stated. Yeast extract peptone dextrose (YPD) medium, buffered glycerol complex (BMGY) medium, and buffered methanol complex (BMMY) medium were prepared according to the manual of Pichia Expression Kit (Version F, Invitrogen). Fermentation Basal Salts Medium (BSM) and PTM1 Trace Salts used for fermentation were prepared according to the Pichia Fermentation Process Guidelines (Invitrogen).

### 2.2. Codon Optimization and Screening of High Xylanase Activity Clones

#### 2.2.1. Codon Optimization and Synthesis of the Gene

The codon usage of* xynB* gene (GenBank DQ302412) from* A. usamii* and* VHb* gene (GenBank AY278220) was analysed using Graphical Codon Usage Analyser (http://gcua.schoedl.de/) and they were optimized by replacing the codons predicted to be less frequently used in* P. pastoris* with the frequently used ones (https://www.dna20.com/). The optimized genes (*xynB-opt* and* VHb*) were synthesized by Sangon (Shanghai, China).

#### 2.2.2. Vector Construction, Transformation, and Screening of High Xylanase Activity Clones

The synthetic gene encoding the mature region of xylanase without the predicted signal sequence was digested by EcoRI and NotI and then ligated into pPICZ*α*A to form pPICZ*α*A*-xynB-opt*. The native xylanase gene (*xynB*) was cloned from* A. usamii* by reverse transcription PCR and then inserted into plasmid pMD20-T to form pMD20-T-*xynB*. The pMD20-T-*xynB *was digested by EcoRI and NotI and then ligated into pPICZ*α*A, resulting in the recombinant plasmid pPICZ*α*A-*xynB*. The synthetic* VHb *gene was digested by EcoRI and NotI and then ligated into pPIC3.5K to form pPIC3.5K-*VHb*. The recombinant plasmids were checked by DNA sequencing.

*P. pastoris* X33 was transformed with 10 *μ*g of PmeI-linearized pPICZ*α*A-*xynB-opt* and pPICZ*α*A-*xynB* vector by electrotransformation, according to Invitrogen's recommendations. Transformants were plated on YPDS plates (10 g/L yeast extract, 20 g/L peptone, 20 g/L dextrose, 20 g/L agar, and 1 M sorbitol) containing 100 *μ*g/mL Zeocin to isolate resistant clones. The method for screening transformants was the same as our previous described method except the microplates [16]. In this study, transformants were picked and cultured in 24-deep-well microplates containing 1.2 mL/well BMGY medium at 30°C for 24 h. After this, the cells were harvested by centrifugation, resuspended, and cultured in 1 mL/well BMMY medium. After 24 h, plates were subjected to centrifugation again and supernatants were used in subsequent activity assays. The clones showing higher activities were checked by shaking flask fermentation.

#### 2.2.3. Expression of* xynB-opt* and* xynB* in* P. pastoris* Shake-Flask Cultures

Thirty clones which had higher enzyme activity were selected for shake-flask cultures. The seeds were inoculated in 10 mL of BMGY medium in a 100 mL shake flask and incubated at 30°C until the culture reached OD_600_ = 2.0–6.0. The cells were harvested by centrifugation and resuspended in 50 mL of BMMY medium and incubated at 30°C. The methanol induction temperature was set at 30°C, and 0.7% (v/v) methanol was fed at 24 h intervals for 5 days. The activities of the xylanase were checked at 24, 48, 72, 96, and 120 h. The colony with the highest activity was selected as the host for the transformation of the pPIC3.5K-*VHb* and pPIC3.5K vectors.

### 2.3. Intracellular Coexpression of VHb

#### 2.3.1. Construction of Recombinant Strains Containing VHb and* xynB-opt*

A clone named X33/*xynB-opt* (the recombinant strain containing* xynB-opt*) exhibiting the maximum xylanase activity under shake-flask cultures was chosen as the host for the transformation of the pPIC3.5K-*VHb* and pPIC3.5K vectors. The plasmids pPIC3.5K-*VHb* and pPIC3.5K were both linearized by SacI and transformed into X33/*xynB-opt* to form X33/*xynB-opt-VHb* and X33/*xynB-opt-p*. The X33/*xynB-opt-p* was used as control during the experiments. The transformants were plated on YPDG plates (10 g/L yeast extract, 20 g/L peptone, 20 g/L dextrose, 20 g/L agar, and 1 M sorbitol) containing 4 mg/mL G418 to isolate resistant clones.

#### 2.3.2. Expression of Recombinant Strains in* P. pastoris* Shake-Flask Cultures

The G418-resistant clones were cultivated in shaking flask. The cultivation conditions and medium composition were the same as described above. The clone exhibiting higher enzyme activity in shaking flask was selected for further experiments.

### 2.4. High Cell Density Fermentation

The transformed strains (X33/*xynB*, X33/*xynB-opt*, X33/*xynB-opt-p,* and X33/*xynB-opt-VHb*) showing the highest xylanase activity in shake-flask culture were cultivated in high cell density fermentor. High cell density fermentation was carried out in 50 L bioreactor (Baoxing Co., Shanghai, China). The cultivation conditions and medium composition were the same as the previous described method [17]. The enzyme activity of the supernatant and dry cell weight (DCW) were monitored throughout the cultivation.

### 2.5. Optimization of the Induction Temperature

To investigate the effects of temperature on the production of xylanase of recombinant strain X33/*xynB-opt-VHb*, the induction temperature was optimized in a 50 L bioreactor. During the methanol fed-phase, the temperature was designed in the range of 30°C to 22°C.

### 2.6. Assay of Xylanase Activity, Protein Determination, Cell Viability, Oxygen Uptake, and Dry Cell Weight

Xylanase activity was assayed according to the method described by previous study and with some modification [18]. All enzyme assays, unless otherwise stated, were carried out at 50°C for 30 min in 100 mM acetic acid-sodium acetic acid buffer (pH 5.0). 2 mL basic reaction mixture (containing 1 mL of 1.0% (w/v) oat spelt xylan and 1 mL of a suitably diluted enzyme solution) was incubated at 50°C for 30 min and reducing sugar (xylose) was measured by the dinitrosalicylic acid (DNS) according to the standard method and xylose was used as a standard. One unit of xylanase activity was defined as the amount of enzyme that produced 1 *μ*M reducing sugar from substrate solution per minute under the assay conditions. The protein content was determined according to the Bradford method using BSA as standard. The measurement of cell viability was performed by methylene blue dye exclusion technique as described by the previous study [19]. The oxygen uptake (OUR) was determined according to the previous studies [20, 21]. Cell density was expressed as grams of dry cell weigh (DCW) per liter of broth and was obtained by centrifuging 10 mL samples in a preweighted centrifuge tube at 8000 g for 10 min and washing twice with deionized water, then allowing the pellet to dry at 100°C to constant weight.

## 3. Results and Discussion

### 3.1. Improved Production of* A. usamii* Xylanase in* P. pastoris* by Codon Optimization

**Figure 1:**
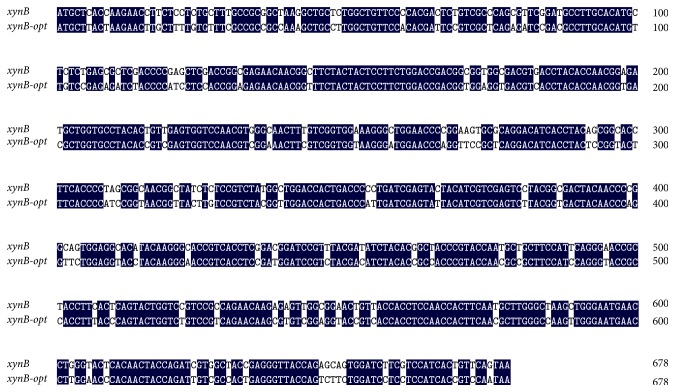
Sequence comparison between the original (*xynB*) and the optimized (*xynB-opt*) genes. Identical residues are marked in black background.

**Figure 2:**
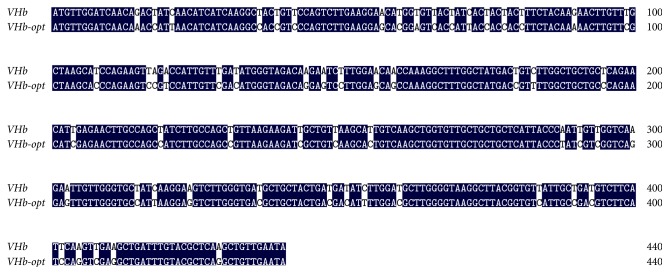
Sequence comparison between the original (*VHb*) and the optimized (*VHb-opt*) genes. Identical residues are marked in black background.

As an easy and simple system,* P. pastoris* is now widely used for heterologous production of recombinant proteins [22]. Due to the discrepancy of codon usage between the host and their original strains, researchers have used codon optimization to increase the expression of heterologous gene. Codon optimization by using frequently used codons in the host is an efficient strategy to improve the expression level of heterologous gene. Generally, this is accomplished by replacing all codons with preferred codons, eliminating AT-rich stretches, and adjusting the G+C content [8, 23]. Analysis of the DNA sequence of* VHb* and native* xynB* using Graphical Codon Usage Analyser revealed that some amino acid residues were encoded by codons that are rarely used in* P. pastoris*. These codons TCG (Ser), CCG (Pro), GGC (Gly), AGC (Ser), and GCG (Ala) shared less than 15% of usage percentage, which may result in a much lower expression level of* xynB* in* P. pastoris* (Tables 1 and 2). To solve this problem, we took a strategy of rewriting the native* xynB* and* VHb* according to* P. pastoris* preferred codon usage. G+C content affects the secondary structure of mRNA and then affects the expression level of heterologous gene. In this study, the G+C content of* VHb* was increased from 42 to 51% and the native* xynB* was reduced from 57.6 to 55.1%, which is in the appropriate range for* Picha* system. Totally, there were 105 and 57 amino acids optimized in native* xynB* and* VHb*, respectively (Tables 1 and 2). As shown in Figures 1 and 2, the optimized gene (*xynB-opt* and* VHb-opt*) shared 83% and 86% of nucleotide sequence identity with that of the native gene (*xynB* and* VHb*).

The recombinant plasmids pPICZ*α*A-*xynB-opt* and pPICZ*α*A-*xynB* were transformed into* P. pastoris* X33 and hundreds of transformants were obtained on YPDZ plates. The positive clones were cultured in 24-deep-well microplates and further screened by xylanase activity assay. Two clones (one carrying* xynB-opt* named X33/*xynB-opt* and the other carrying* xynB* named X33/*xynB*) showing the higher activity were chosen for shake-flask cultures. The recombinant strains X33/*xynB-opt* and X33/*xynB* were cultivated in shaking flask. After 120 h of cultivation under inducing conditions, the xylanase activities of X33/*xynB-opt* and X33/*xynB* were 920 U/mL and 520 U/mL, respectively. The total protein content of X33/*xynB-opt* and X33/*xynB* were 0.25 mg/mL and 0.15 mg/mL, respectively. The recombinant strain X33/*xynB-opt* was chosen as the host for the transformation of the pPIC3.5K-*VHb* and pPIC3.5K vectors.

### 3.2. Intracellular Coexpression of VHb

Oxygen supply is one of the most critical parameters for cell growth and heterologous protein expression in recombinant* P. pastoris*. VHb is a suitable oxygen uptake improving protein for expression in* P. pastoris* due to its high oxygen trapping and releasing ability, enabling it to satisfy extremely high oxygen demand during fermentations [24]. In this study, in order to enhance oxygen uptake and improve the production of recombinant xylanase, we attempted to coexpress the* VHb* with* xynB-opt* in* P. pastoris*. The recombinant plasmids pPIC3.5K-*VHb* and pPIC3.5K were linearized and transformed into recombinant strain X33/*xynB-opt* to form recombinant strains VHb^+^ (X33/*xynB-opt* containing* VHb*) and VHb^−^ (X33/*xynB-opt* containing pPIC3.5K). Transformants were plated on YPDG plates. Then the G418-resistant clones were cultured in shaking flasks. The VHb^+^ (named X33/*xynB-opt-VHb*) showed higher cell density and xylanase activity than VHb^−^ (named X33/*xynB-opt-p*). The cell density of X33/*xynB-opt-VHb* was approximately 2.5 g/L higher than X33/*xynB-opt-p*. Moreover, the xylanase activity of X33/*xynB-opt-VHb* was 1300 U/mL, which was 31% and 60% more than that of X33/*xynB-opt-p *and X33/*xynB*.

### 3.3. High Cell Density Fermentation

**Figure 3:**
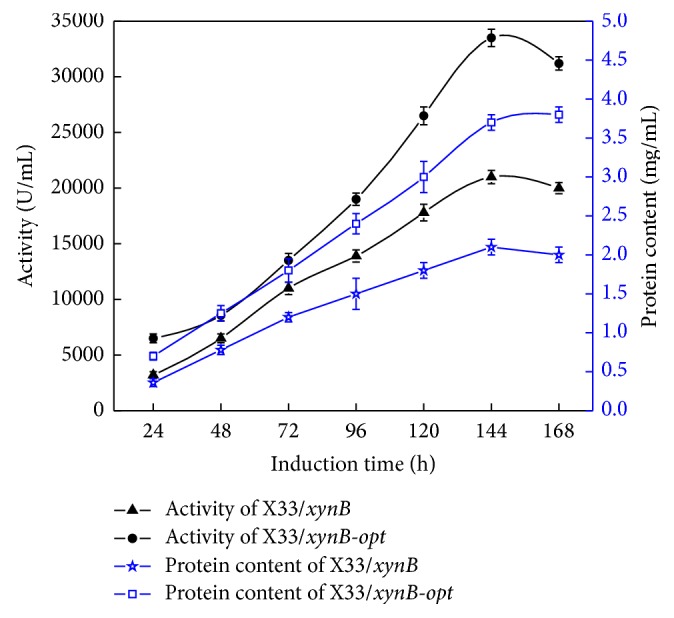
Xylanase activity and total protein content of X33/*xynB-opt* and X33/*xynB* during fed batch fermentation in 50 L bioreactor.

In order to obtain a large amount of active protein, the recombinant strains X33/*xynB-opt*, X33/*xynB*, X33/*xynB-opt-p,* and X33/*xynB-opt-VHb* were cultivated in 50 L fermentor. As shown in Figure 3, the maximum xylanase activity and total protein concentration produced by X33/*xynB-opt* reached 33500 U/mL and 3.8 g/L, respectively. Compared with X33/*xynB*, the maximum xylanase activity and total protein concentration were increased by 60% and 80%, respectively. In this study, the fermentation conditions and DCW of X33/*xynB-opt* and X33/*xynB* were almost the same during the high cell density fermentation (data not shown). These results showed that the improved production of recombinant xylanase in* P. pastoris* was reached by codon optimization. Codon optimization is an effective method to improve the expression level of heterologous gene in* P. pastoris*. In our previous study, the *α*-amylase gene from* Bacillus licheniformis* was codon optimization according to the codon usage of* P. pastoris* and the optimized gene was expressed at a significantly higher level than the wild-type gene [17]. Through codon optimization the glucanase gene from* Fibrobacter succinogenes* resulted in a 2.34-fold increase of target protein production [25].

**Figure 4:**
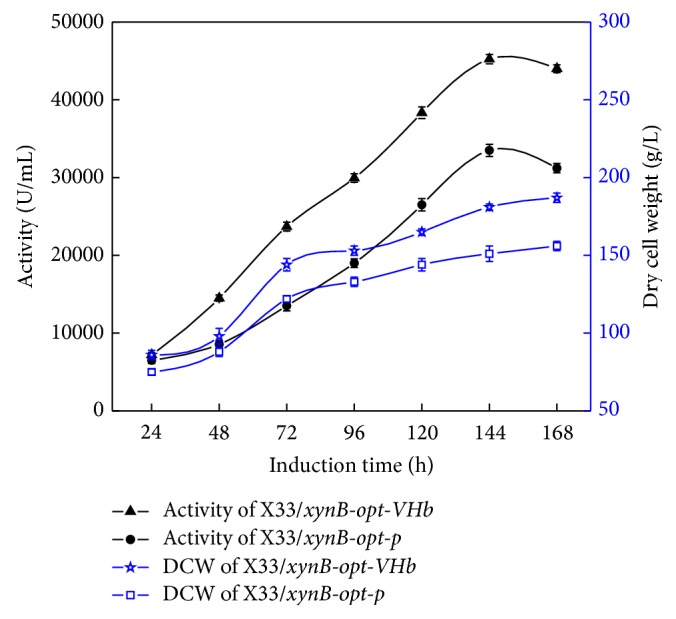
Xylanase activity and DCW of X33/*xynB-opt-p* and X33/*xynB-opt-VHb* during fed batch fermentation in 50 L bioreactor.

**Figure 5:**
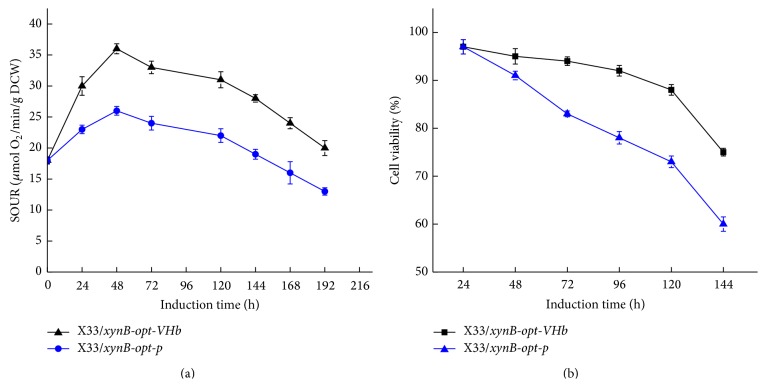
Comparison of specific oxygen uptake (a) and cell viability (b) profile between X33/*xynB-opt-p* and X33/*xynB-opt-VHb* during fed batch fermentation in 50 L bioreactor.

The growth and xylanase activity profile of X33/*xynB-opt-p* and X33/*xynB-opt-VHb* in 50 L bioreactor were shown in Figure 4. The DCW and xylanase activity of X33/*xynB-opt-VHb* were higher than those of X33/*xynB-opt-p* during the methanol induction phase. The highest xylanase activity of X33/*xynB-opt-VHb* was 45225 U/mL which was about 1.35-fold higher than that of X33/*xynB-opt-p*. The higher xylanase activity and DCW of X33/*xynB-opt-VHb* were probably caused by coexpression of VHb. The function of VHb is usually considered to be the enhancement of respiration, cell viability, and energy metabolism by facilitating oxygen uptake. As shown in Figure 5(a), SOUR of both strains was almost the same before methanol induction. After induction, SOUR of X33/*xynB-opt-VHb* increased higher than X33/*xynB-opt-p* during the whole induction phase, which was probably caused by VHb expression improving oxygen utilization and respiratory efficiency. The VHb-expressing strain with higher oxygen demand was similar to previous studies [26]. Furthermore, we also compared the cell viabilities of X33/*xynB-opt-p* and X33/*xynB-opt-VHb*. The cell viabilities of X33/*xynB-opt-p* and X33/*xynB-opt-VHb* at the end of 144 h of cultivation were 75% and 60%, respectively (Figure 5(b)). This indicates that VHb expression resulted in an increase in cell viability. In this study, coexpression of VHb increased SOUR and then improved cell viability and DCW of X33/*xynB-opt-VHb*. Our results indicated that coexpression of VHb is also an effective method to improve the production of heterologous protein in* P. pastoris*.

### 3.4. Optimization of the Induction Temperature

**Figure 6:**
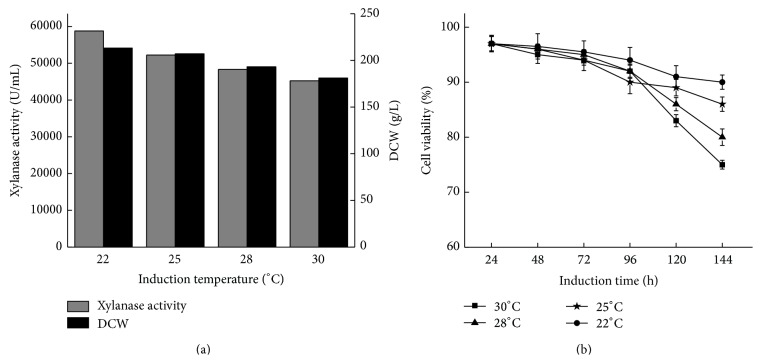
Xylanase activity and DCW of X33/*xynB-opt-VHb* during fed batch fermentation in 50 L bioreactor under different induction temperature (a). Cell viability profile of X33/*xynB-opt-VHb* during fed batch fermentation in 50 L bioreactor under different induction temperature (b).

Temperature is a key factor for optimization of heterologous proteins expressed in* P. pastoris*. In order to evaluate the effects of cultivation temperature on cell growth, cell viability, and xylanase production, X33/*xynB-opt-VHb* was grown in BSM (pH 5.0) at 22, 25, 28, and 30°C (Figure 6). As shown in Figure 6(a), xylanase activities at lower induction temperature were higher than that at higher induction temperature. The maximum xylanase activity of 58792 U/mL with a cell density of 213 g DCW was obtained after 144 h of culture at 22°C, which was 1.29-fold and 1.17-fold higher than that at 30°C. Until now, several* Aspergillus* endo-*β*-1,4-xylanases have also been successfully expressed in* P. pastoris*. The* Aspergillus sulphureus* and* Aspergillus niger* endo-*β*-1,4-xylanases were functionally expressed and secreted in the* P. pastoris*, the enzyme activity of which reached 105 and 20424 U/mL [8, 27], which were lower than the expression level of* A. usamii* endo-*β*-1,4-xylanase in this study. However, these values are not fully comparable since different cultivation conditions and activity assays with different substrates and conditions have been used. Meanwhile, the cell viabilities of X33/*xynB-opt-VHb* under different temperature were also determined. The cell viability remained below 80% under the temperature at 30°C and 28°C and above 90% in the cultivation with a temperature of 22°C (Figure 6(b)). These results indicated that lower induction temperature could facilitate production of recombinant xylanase. According to the findings by other researchers, lowering induction temperature can reduce cell death and increase cell viability and then improved the production of heterologous proteins in* P. pastoris *[28]. Furthermore, lowering induction temperature can enlarge VHb effect on cell performance of* P. pastoris* and then obtain a higher final cell density and viability in comparison with higher temperature [24].

## 4. Conclusions

In this study, we combined codon optimization, intracellular coexpression of VHb, and optimization of induction temperature as a whole to improve the expression level of recombinant* A. usamii* endo-*β*-1,4-xylanase in* P. pastoris*. To our knowledge, this is the first report to combine these methods as a whole to improve the production of* A. usamii* endo-*β*-1,4-xylanase in* P. pastoris*. Our results indicated that combined codon optimization, intracellular coexpression of VHb, and optimization of induction temperature are an effective method to improve the production of heterologous protein in* P. pastoris*. Furthermore, our results presented here will greatly contribute to improving the production of recombinant proteins in* P. pastoris *and offer a greater value in various industrial applications.

## Figures and Tables

**Table 1:** Comparison of the codon usage for native (*xynB*) and synthetic (*xynB-opt*) targeted genes  at *Pichia pastoris* for expression.

AA	Codon	Host fraction	*xynB*	*xynB-opt*
Gly	GGG	0.10	0	0
	GGA	0.32	9	11
	GGT	0.44	5	18
	GGC	0.14	15	0

Glu	GAG	0.43	6	6
	GAA	0.57	0	0

Asp	GAT	0.58	3	2
	GAC	0.42	7	8

Val	GTG	0.19	3	0
	GTA	0.15	0	0
	GTT	0.42	6	1
	GTC	0.23	6	14

Ala	GCG	0.06	2	0
	GCA	0.23	0	0
	GCT	0.45	12	7
	GCC	0.26	4	11

Arg	AGG	0.16	0	0
	AGA	0.48	1	2
	CGG	0.05	0	0
	CGA	0.10	0	0
	CGT	0.16	2	3
	CGC	0.05	2	0

Lys	AAG	0.53	6	5
	AAA	0.47	0	1

Ser	AGT	0.15	3	0
	AGC	0.09	4	0
	TCG	0.09	5	0
	TCA	0.19	0	0
	TCT	0.29	3	6
	TCC	0.20	9	18

Stop	TAA	0.53	1	1

Asn	AAT	0.49	2	0
	AAC	0.51	13	15

Met	ATG	1.00	3	3

Ile	ATA	0.19	0	0
	ATT	0.50	1	1
	ATC	0.30	6	6

Thr	ACG	0.11	1	0
	ACA	0.24	1	0
	ACT	0.40	4	4
	ACC	0.25	19	23

Trp	TGG	1.00	6	6

Cys	TGT	0.65	0	1
	TGC	0.35	1	0

Tyr	TAT	0.46	2	1
	TAC	0.55	15	16

Leu	TTG	0.33	1	8
	TTA	0.16	0	0
	CTG	0.16	4	0
	CTA	0.11	0	0
	CTT	0.16	1	3
	CTC	0.08	5	0

Phe	TTT	0.54	2	1
	TTC	0.46	5	6

Gln	CAG	0.39	8	7
	CAA	0.61	0	1

His	CAT	0.57	0	0
	CAC	0.43	4	4

Pro	CCC	0.15	4	0
	CCG	0.09	1	0
	CCA	0.41	0	6
	CCT	0.35	1	0

**Table 2:** Comparison of the codon usage for native (*VHb*) and synthetic (*VHb-opt*) targeted genes  at *Pichia pastoris *for expression.

AA	Codon	Host fraction	*VHb*	*VHb-opt*
Gly	GGG	0.10	0	0
	GGA	0.32	1	0
	GGT	0.44	7	8
	GGC	0.14	0	0

Glu	GAG	0.43	7	1
	GAA	0.57	2	8

Asp	GAT	0.58	2	8
	GAC	0.42	6	0

Val	GTG	0.19	0	0
	GTA	0.15	0	0
	GTT	0.42	4	10
	GTC	0.23	10	4

Ala	GCG	0.06	0	0
	GCA	0.23	0	0
	GCT	0.45	17	23
	GCC	0.26	6	0

Arg	AGG	0.16	0	0
	AGA	0.48	1	2
	CGG	0.05	0	0
	CGA	0.10	0	0
	CGT	0.16	1	0
	CGC	0.05	0	0

Lys	AAG	0.53	9	10
	AAA	0.47	1	0

Ser	AGT	0.15	0	0
	AGC	0.09	0	0
	TCG	0.09	0	0
	TCA	0.19	0	0
	TCT	0.29	0	1
	TCC	0.20	1	0

Stop	TAA	0.53	1	1

Asn	AAT	0.49	0	0
	AAC	0.51	4	4

Met	ATG	1.00	3	3

Ile	ATA	0.19	0	0
	ATT	0.50	5	5
	ATC	0.30	7	7

Thr	ACG	0.11	0	0
	ACA	0.24	0	0
	ACT	0.40	1	8
	ACC	0.25	7	0

Trp	TGG	1.00	1	1

Cys	TGT	0.65	1	1
	TGC	0.35	0	0

Tyr	TAT	0.46	0	0
	TAC	0.55	4	4

Leu	TTG	0.33	14	14
	TTA	0.16	0	0
	CTG	0.16	0	0
	CTA	0.11	0	0
	CTT	0.16	0	0
	CTC	0.08	0	0

Phe	TTT	0.54	0	2
	TTC	0.46	4	2

Gln	CAG	0.39	6	2
	CAA	0.61	3	7

His	CAT	0.57	1	4
	CAC	0.43	3	0

Pro	CCC	0.15	0	0
	CCG	0.09	0	0
	CCA	0.41	6	7
	CCT	0.35	1	0
